# Statistical learning of spatiotemporal target regularities in the absence of saliency

**DOI:** 10.3758/s13414-024-02992-6

**Published:** 2025-01-09

**Authors:** Zhenzhen Xu, Jan Theeuwes, Sander A. Los

**Affiliations:** 1 Department of Experimental and Applied Psychology, Vrije Universiteit Amsterdam, Van Der Boechorststraat 7, 1081 BT Amsterdam, The Netherlands; 2 Institute Brain and Behavior Amsterdam (iBBA), Amsterdam, The Netherlands

**Keywords:** Visual attention, Spatiotemporal regularities, Visual statistical learning, Bottom-up attention

## Abstract

In previous studies, it was established that individuals can implicitly learn spatiotemporal regularities related to how the distribution of target locations unfolds across the time course of a single trial. However, these regularities were tied to the appearance of salient targets that are known to capture attention in a bottom-up way. The current study investigated whether the saliency of target is necessary for this type of learning to occur. In a visual search task, participants were instructed to search for a unique circle with a gap (Landolt C) among two other circles and indicate the location of the gap. Unbeknownst to them, the onset timing of search displays predicted the target location. Specifically, the target appeared more frequently at one peripheral location with an early onset of the search display and at the opposite peripheral location with a late onset of the search display. Additionally, we manipulated the gap size of the Landolt C to create either nonsalient (small gap) or salient (big gap) target events. Results showed that, regardless of prior exposure to salient targets (Experiment 1) or nonsalient targets (Experiment 2), visual search efficiency increased when the target appeared at the temporally valid location compared with the temporally invalid location. In conclusion, the saliency of targets and the associated bottom-up capture is not a prerequisite for learning dynamic distributional regularities of target locations during visual search.

**Supplementary Information:**

The online version contains supplementary material available at 10.3758/s13414-024-02992-6.

In our daily life, we effortlessly learn the statistical patterns associated with various activities through repeated engagement. Imagine a scenario of using a printer: With consistent practice, we come to anticipate that, upon pressing the print button, the printed output will emerge after a certain temporal delay from a specific place in the printer. This ability to learn the typical spatiotemporal regularities associated with printing allows us to efficiently interact with the printer. Such learning reflects a form of unconscious and automatic visual learning process known as visual statistical learning (VSL), where the visual system becomes skilled at recognizing the statistical patterns and regularities that it encounters within the visual stream without explicit awareness (Fiser & Aslin, 2001; Turk-Browne et al., 2005).

## Visual statistical learning in space

Through past search experiences, the visual system can incidentally learn the spatial arrangements of our surroundings. In visual streams, the occurrence of certain objects in space may exhibit an imbalance when the search goal could be found more often at specific locations than at others (Geng & Behrmann, 2002, 2005). It has been shown that individuals are able to implicitly learn these probable target locations. With increased attention directed towards these learned locations, visual search efficiency improves when the target subsequently appears there (Geng & Behrmann, 2002, 2005). Notably, this capability to learn the spatial distribution is not confined to task-relevant objects. Even in the case of a task-irrelevant salient object, individuals can learn its likely location during the search for the target. By suppressing the location that is most likely to contain a distractor, the distractor causes less interference when searching for the target (for a review, see Theeuwes et al., 2022; Wang & Theeuwes, 2018). Beyond these straightforward spatial distributions of individual objects, individuals are also able to learn the recurring spatial arrangements of multiple objects (Chun & Jiang, 1998; Fiser & Aslin, 2001, 2002).

## Visual statistical learning in time

While we can learn to anticipate where in our environment relevant information may appear, the visual surroundings are often dynamic and can change over time. In visual search, it is also of great importance to predict when an object might appear (Coull & Nobre, 1998; Nobre & van Ede, 2018). Building on past search experiences, the visual system can also incidentally learn the temporal aspects of the surroundings. Alongside the spatial distribution of objects, the occurrence of visual elements can vary with the passage of time. For instance, a visual object may appear more frequently at certain time points than at other time points. Studies investigating time often manipulate the distribution of intervals (or foreperiods) between a neutral warning cue and a subsequent target stimulus to induce different temporal expectations of target onset (Niemi & Näätänen, 1981). Consequently, the target may appear more frequently either after a short interval or after a long interval. Research indicates that individuals can incidentally learn the temporal distribution of visual objects during search tasks, leading to optimized perceptual processing and enhanced motor preparation at time points with a high probability of target occurrence (Los et al., 2017; Näätänen, 1971; Salet et al., 2022; Trillenberg et al., 2000; Vangkilde et al., 2012, 2013).

## Visual statistical learning of space unfolding in time

The preceding research findings highlight individuals’ ability to learn spatial or temporal regularities of visual objects through consistent search experiences. However, real-life visual environments often involve more complex scenarios where spatial arrangements of objects dynamically evolve over time. In such situations, visual inputs could also contain repeated spatiotemporal patterns within and across visual events.

Within a single visual event, the spatial distribution of objects may vary across different time points. Xu et al. (2023) showed implicit learning of a dynamic spatial distribution of targets unfolding at varying time points of a trial. In a variant of the additional singleton task (Theeuwes, 1991, 1992), participants were instructed to search for a pop-out target singleton with a unique shape among five nontargets. Unbeknownst to them, the target location was manipulated based on the duration of the interval preceding the onset of the target. Specifically, the target appeared more frequently at one location after a short interval and at the opposite location after a long interval. The results demonstrated faster visual search when the target appeared at the location after its associated interval than when it occurred there after a nonassociated interval, while participants remained unaware of these regularities. This indicates that participants incidentally extracted the probable target location at different time points of a trial. A proposed mechanism behind this learning process was the dynamic allocation of spatial attention within a trial, adapting as time progressed.

This form of spatiotemporal learning has also been demonstrated in dynamic environments with greater noise. Boettcher et al. (2022) introduced a dynamic search task in which targets and nontargets unfolded over time at various spatial locations. Participants were asked to search for and click on eight instances of a target (vertical line) that faded in and out of a display and to ignore similarly transient distractors (tilted lines) on a noisy background. On each trial, four of the eight targets appeared at predictable times and locations. For instance, the target could appear more often at the lower left quadrant at one time point and more often at the upper left quadrant at another time point. The other four targets appeared completely unpredictably in both space and time. The results revealed enhanced performance for spatiotemporally predictable targets compared with unpredictable ones, showcasing the adaptability of individuals to learn and apply spatiotemporal regularities even in dynamic and noisy settings that are more representative of real-life surroundings.

In addition to distributional spatiotemporal regularities within individual visual events, the encountered spatial inputs may exhibit repeated patterns across successive events. These transitional regularities, observed over sequential spatial arrays, introduce spatiotemporal predictability beyond individual occurrences. In this context, the spatial arrangements in one visual event become predictive of the next. Li and Theeuwes (2020) demonstrated the learning of intertrial statistical associations regarding target locations. Using the additional singleton task, participants unknowingly encountered two sets of temporal sequences governing target locations across trials. In certain instances, the target location in a given trial predicted the target location in the subsequent trial with 100% validity (e.g., left-right; bottom-top). Despite participants lacking awareness of these regularities, visual search performance was enhanced when the target appeared at a predicted location compared with a nonpredicted location.

This remarkable adaptability of individuals to the cross-trial transitions in target location was replicated even when transitioning from deterministic to probabilistic conditions (Yu et al., 2023). However, replicating exact across-trial target-to-target location regularities in Li and Theeuwes (2020) proved challenging in a *T*-among-*L* search task (Li et al., 2022), where participants searched for a grey letter *T* among grey letters *L*. Intriguingly, introducing a salient color to make the target *T* stand out facilitated the learning of these regularities. And this acquired bias persisted even when the target later lost its saliency. These varying findings raise questions about the conditions under which individuals can learn sequence-based spatiotemporal regularities. The saliency of the target emerges as a crucial factor. When targets lack saliency, the noise introduced by searching among nontargets may impede the detection of across-trial associations. Conversely, with salient targets, immediate attentional capture by the target allows for learning across-trial target locations (Li et al., 2022; Ono et al., 2005).

## The current study

While the significance of target saliency in acquiring sequence-based spatiotemporal regularities is established, it remains unclear whether this factor is critical for learning interval-based spatiotemporal regularities. Currently, evidence supporting VSL of interval-based spatiotemporal target regularities primarily involves salient targets that stand out from the background (Wagener & Hoffmann, 2010; Xu et al., 2023). Therefore, the learning could be fueled by the immediate bottom-up attentional capture of targets driven by its saliency. While Boettcher et al. (2022) reported learning of spatiotemporal distributions of a less conspicuous target within a noisy background, the need to click on the detected targets introduces the possibility that motor learning contributed to the observed effect. Consequently, the question of whether VSL of interval-based spatiotemporal target regularities necessitates the saliency of stimuli remains unresolved.

The current study aimed to investigate whether saliency is a prerequisite for learning distributional spatiotemporal target regularities while mitigating potential motor response learning. In two online experiments, we presented participants with a horizontal array, containing a Landolt C (target), with its gap at the top or at the bottom, among two full circles (distractors). Participants were asked to search for the Landolt C and respond to the location of its gap. Crucially, the probability that the target appeared left or right in the array was associated with the duration of the preceding interval. That is, one peripheral location had a high probability of containing the target after the short interval (500 ms), while the other peripheral location had a high probability after the long interval (1,500 ms). As the response feature (up or down) was independent of the target location (left or right), we could exclude a motor response learning interpretation of possible effects. If participants learned these relationships, they should allocate their attention to the high probability location at the associated time point, thereby enhancing performance.

To examine the role of saliency in learning these spatiotemporal regularities, we further manipulated target saliency between experiments. Typically, bottom-up saliency arises from the feature contrast of a stimulus relative to its local surrounding in low-level visual features such as color, luminance, orientation, size, or motion. This contrast makes the stimulus stand out from its surroundings, automatically capturing attention (Itti & Koch, 2001; Wolfe & Horowitz, 2004, 2017). The saliency of a target increases with its difference from distractors and with the homogeneity of the distractors along basic feature dimensions (Duncan & Humphreys, 1989; Wolfe & Horowitz, 2017). In the present study, we manipulated target saliency by altering difference between the target Landolt C and the nontarget circles. Specifically, we varied the gap size of the target Landolt C relative to nontarget circles. In Experiment 1, the gap was either big (44 pixels) or small (2 pixels). The Landolt C with a big gap stands out more from nontarget circles compared with one with a small gap, making the target more salient to observers. In turn, it should capture attention more easily and lead to better detection. In Experiment 2, the gap size was consistently small (2 pixels). If participants can learn the regularities regardless of target saliency, then saliency is not a prerequisite for learning these relations.

## Experiment 1

In Experiment 1, we manipulated target saliency in different blocks to examine a possible modifying effect on learning spatiotemporal target regularities. In the initial two blocks (Blocks 1–2), the Landolt C featured a large gap, thus shaping a salient target relative to nontargets (circles). Subsequently, in Blocks 3–5, a deliberate reduction in the gap size transformed the targets into nonsalient targets.

According to previous research on transitional spatiotemporal regularities (Li & Theeuwes, 2020; Li et al., 2022), participants can easily learn the regularities when the target is salient and stands out from the nontargets, often showing learning effects within the first block. Conversely, when the target is nonsalient and blends in with the nontargets, participants are unable to learn these regularities, unless the regularities are initially introduced containing a salient feature (Li et al., 2022). That is, the learning effect from exposure to a pop-out target persists through subsequent blocks with nonsalient targets. Based on these findings, we anticipated that participants would learn and adapt easily to the distributional spatiotemporal regularities in the two blocks with the big-gap condition due to its high target saliency. In the subsequent three blocks with the small-gap condition, we examined whether learned spatiotemporal regularities would persist even when the target was no longer salient.

### Method

#### Participants

The sample size was determined by a prior power analysis using the *simr* package (Green & MacLeod, 2016; Version 1.0.7). Based on the data (*N* = 41) from a previous study (Xu et al., 2023), we derived the effect size for the interaction of interval (500 ms, 1,500 ms) and target location probability (high_early, high_late). To mitigate the risk of conducting analyses based on inflated effect sizes from published data (Kumle et al., 2021), we scaled down the effect size to 90% of the original size. The analysis based on 1,000 simulations indicated that a sample size of 55 participants would yield a power of 0.82 to detect the interaction effect. Fifty-five students were recruited from Prolific, and one participant was excluded due to low overall accuracy (< 70%). The final dataset consisted of 54 participants (*M* = 23.59 years, *SD* = 3.01, 34 women, 19 men, one other).

This study received approval from the Ethical Review Committee of the Faculty of Behavioral and Movement Sciences at Vrije Universiteit Amsterdam. Informed consent was obtained from all participants at the beginning of the experiment, and upon completion, participants were compensated with monetary payment.

#### Apparatus and stimuli

This experiment was developed in OpenSesame (Version 3.3.11; Mathôt et al., 2012) using OSWeb 1.4.4.0, and implemented online through JATOS (Lange et al., 2015). Participants were instructed to conduct the experiment in a quiet environment on a laptop or desktop.

**Fig. 1:**
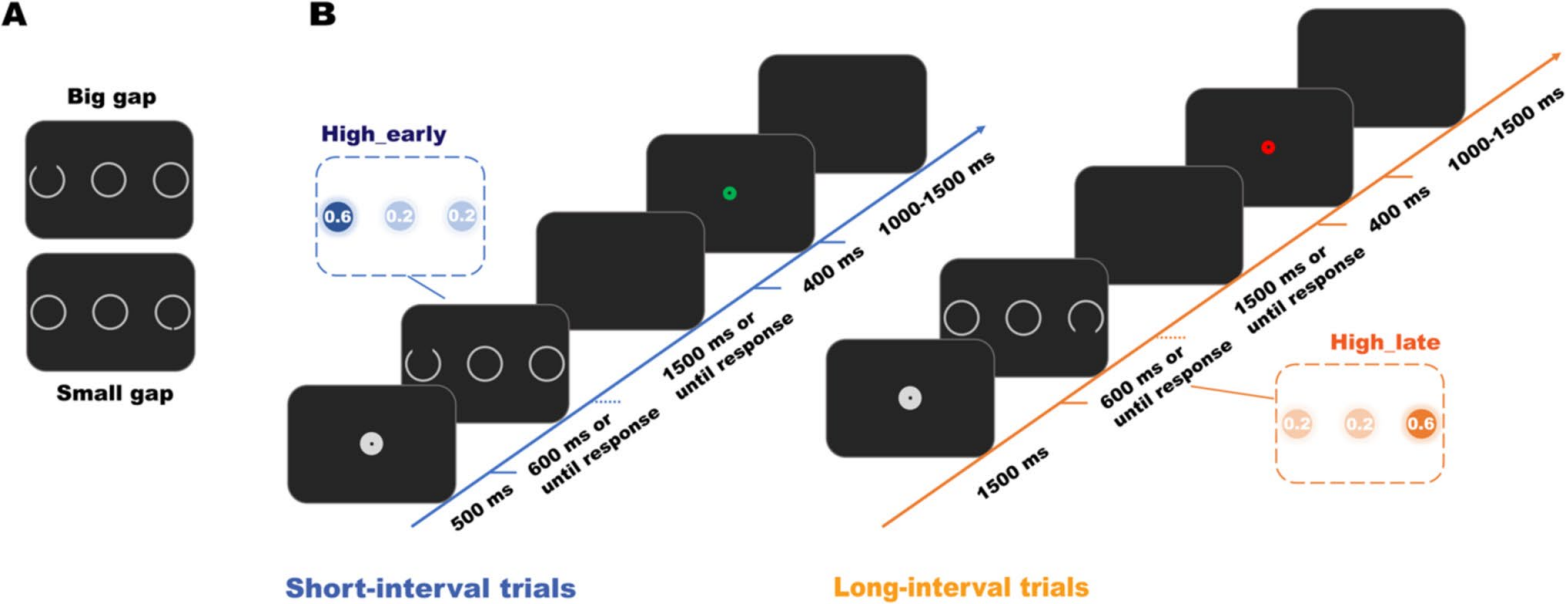
The stimuli and trial sequences of Experiment 1. **A** Sample search displays with a big-gap (Blocks 1–2) and a small-gap (Blocks 3–5) target in Experiment 1. **B** The sample trial sequences for short-interval trials (blue timeline) and long-interval trials (orange timeline). Following a short (500 ms) or long (1,500 ms) fixation period, the search display appeared, which required participants to indicate whether the target (the Landolt C) exhibited an opening at the top or bottom. The target occurred with higher probability at either the left or right location depending on the duration of the interval, resulting in a “high_early” and “high_late” location (insets show probabilities of occurrence). After the response, feedback was promptly presented for 400 ms. Stimuli are not drawn to scale. (Color figure online)

The stimuli (Fig. 1) were light-grey objects (#d1d1d1) presented against a black background. The fixation display featured a circle with a 16-pixel radius and a 2-pixel hole at the center. The search display comprised three circles with a 54-pixel radius and 7-pixel grey outline. One circle was centrally located, while the other two appeared in the periphery, positioned 864 pixels away on the left and right side of the central location. The target, uniquely identifiable by a gap at either the top or bottom, took the form of a Landolt C. The gap size of the target varied based on the block number, either 44 pixels (big-gap) or 2 pixels (small-gap), as illustrated in Fig. 1A. The feedback display featured a green (correct) or red (incorrect or missed response) circle, each with an 8-pixel radius and a 2-pixels hole.

#### Procedure and design

As shown in Fig. 1B, each trial started with a fixation dot, on which participants were required to fixate. Following an equiprobable interval of 500 or 1,500 ms, a search display appeared for a maximum duration of 600 ms, which was then replaced by a blank display. Participants had to search for the Landolt C and indicate the location of the gap by pressing the “H” key (top) or “B” key (bottom) with their right or left index finger, respectively. They were instructed to initiate the search from the central location and to respond as quickly and accurately as possible. The maximum response window was 2,100 ms, commencing from the onset of the search display. Following the participant’s response or the expiration of the response window, the feedback display was presented for 400 ms. After feedback offset, a blank screen was presented for a duration that jittered between 1,000 and 1,500 ms.

The experiment manipulated the association between the interval and target location, as outlined in Table 1. Specifically, for a random half of the participants, the target had a probability of 0.60 to appear at the left location following the 500-ms interval (high_early location) and at the right location following the 1,500-ms interval (high_late location), while this probability was 0.20 for the other two locations after either interval. For the other half of the participants, this association was reversed. The gap of the Landolt C occurred equally often at the top or bottom under all conditions. Given these constraints, all conditions were randomized within each block of 160 trials.

**Table 1:** Trial distribution across experimental conditions in each block

Target location	High_early	High_late	Central		Total
Interval (ms)					
500	48	16		16	80
1,500	16	48		16	80
Total	64	64		32	160

In the practice block and the initial two experimental blocks (Blocks 1–2), the salient target with a large gap size was used, which made the target stand out from other nontarget circles. After the completion of the initial two experimental blocks, participants were informed that the gap size would diminish in the subsequent three blocks. During Blocks 3–5, the nonsalient target was used, whose small gap made it less distinguishable from the nontarget circles and harder to detect, especially in peripheral positions.

The experiment started with a practice block of 24 trials. In the practice block, there was no association between the interval and target location, such that the target appeared equally often in each of the three locations in every interval. Only upon achieving an overall accuracy of 65% during practice did participants advance to the formal experiment, which included five blocks of 160 trials each. During the experiment, a rest display was presented midway and at the end of each block to prompt participants to take breaks. Feedback on performance (accuracy and mean response time [RT]) was provided at the end of each block. Upon completion of the fifth experimental block, participants answered questions to estimate their awareness of the spatiotemporal distribution of the target. First, they were asked to indicate whether they noticed the interval variations across trials by providing a simple yes/no response. Next, they were prompted to identify the most frequently occurring target location following the short and long interval, respectively.

#### Data analyses

Trials with RT below 200 ms or above 2,200 ms (0.17% of all trials) were excluded. To further refine the RT analysis, a two-step data-trimming procedure was implemented. Firstly, incorrect trials (8.67% of all trials) were excluded. Secondly, trials with RTs beyond ± 2.5 standard deviations from the mean of each target location for each participant (2.31% of all trials) were also excluded.

Linear mixed-effects models (LMMs) were employed for the analysis of RTs, while generalized linear mixed-effects models ((G)LMMs) with a binomial distribution were utilized for accuracy (ACC) as a binary response. The choice of mixed-effects models was motivated by their suitability for handling the unbalanced experimental design with varying trial numbers across different conditions (Baayen et al., 2008; Jaeger, 2008), a characteristic applicable to the current study. The analyses were executed using the *lme4* package (Version 1.1–33; Bates et al., 2015) in RStudio (Version 2023.03.0 + 386; RStudio Team, 2023). In (G)LMMs, all fixed-effects factors were dummy coded, and the random-effects structures were determined by running the maximal random-effects structure justified by the design, which allowed model convergence (Barr et al., 2013).

First, we compared performance across the three target locations. In the (G)LMMs, the fixed factor included the target location (high_early, high_late, central), while the random-effect structure comprised a by-participant random intercept and a by-participant random slope for the target location. The *p* value of the fixed effect was obtained using the *ImerTest* package (Version 3.1–3; Kuznetsova et al., 2017) with degrees of freedom estimated by Satterthwaite approximation. To discern specific differences, pairwise comparisons were implemented using the *emmeans* package (Version 1.10.2; Lenth, 2024), with the Tukey correction for *p*-value adjustment when comparing multiple estimates within a family of comparisons.

Next, we exclusively compared performance in the peripheral locations at different time points across phases with varying target saliencies. In these analyses, (G)LMMs incorporated fixed effects of interval (500 ms, 1500 ms), target location (high_early, high_late), phase (blocks 1–2, blocks 3–5), and the three-way interaction. Recognizing potential contributions from short-term intertrial priming (Maljkovic & Nakayama, 1994) and participants’ awareness of the regularities (Vadillo et al., 2020), control fixed-effects factors were also introduced in the models. These factors included intertrial target location priming (repeated, nonrepeated), intertrial interval priming (repeated, nonrepeated), intertrial response priming (repeated, nonrepeated), as well as participants’ awareness (as evidenced by the query) of the probable target location at the early (aware, unaware) or late time point (aware, unaware). Within these (G)LMMs, the random effect structure featured a by-participant random intercept and a by-participant random slope for target location, interval, and block phase. Likelihood ratio tests were employed to obtain *p* values for the interactions in all model comparisons, where the model with the fixed effect of interest was compared with the model without it (α = 0.05).

Further, we examined the two-way interaction of interval and target location for each phase with different levels of target saliency. In the (G)LMMs, the models incorporated fixed effects of interval, target location, the interaction of interval and target location, and all the control fixed-effects factors. The random-effect structure of the LMMs comprised a by-participant random intercept and a by-participant random slope for the interval, target location, and their interaction. In the (G)LMMs, the random-effect structure featured a by-participant random intercept and a by-participant random slope for the interval.

Lastly, we conducted a block-by-block analysis to investigate the time course of learning across blocks (see Appendix A for details), acknowledging the potential limitations in statistical power. First, we examined the three-way interaction of interval (500 ms, 1,500 ms), target location (high_early, high_late), and block number (1–5) using (G)LMMs. Following the significant three-way interaction, we then investigated the two-way interaction between interval and target location within each block separately.

### Results

#### Overall performance across locations

**Fig. 2:**
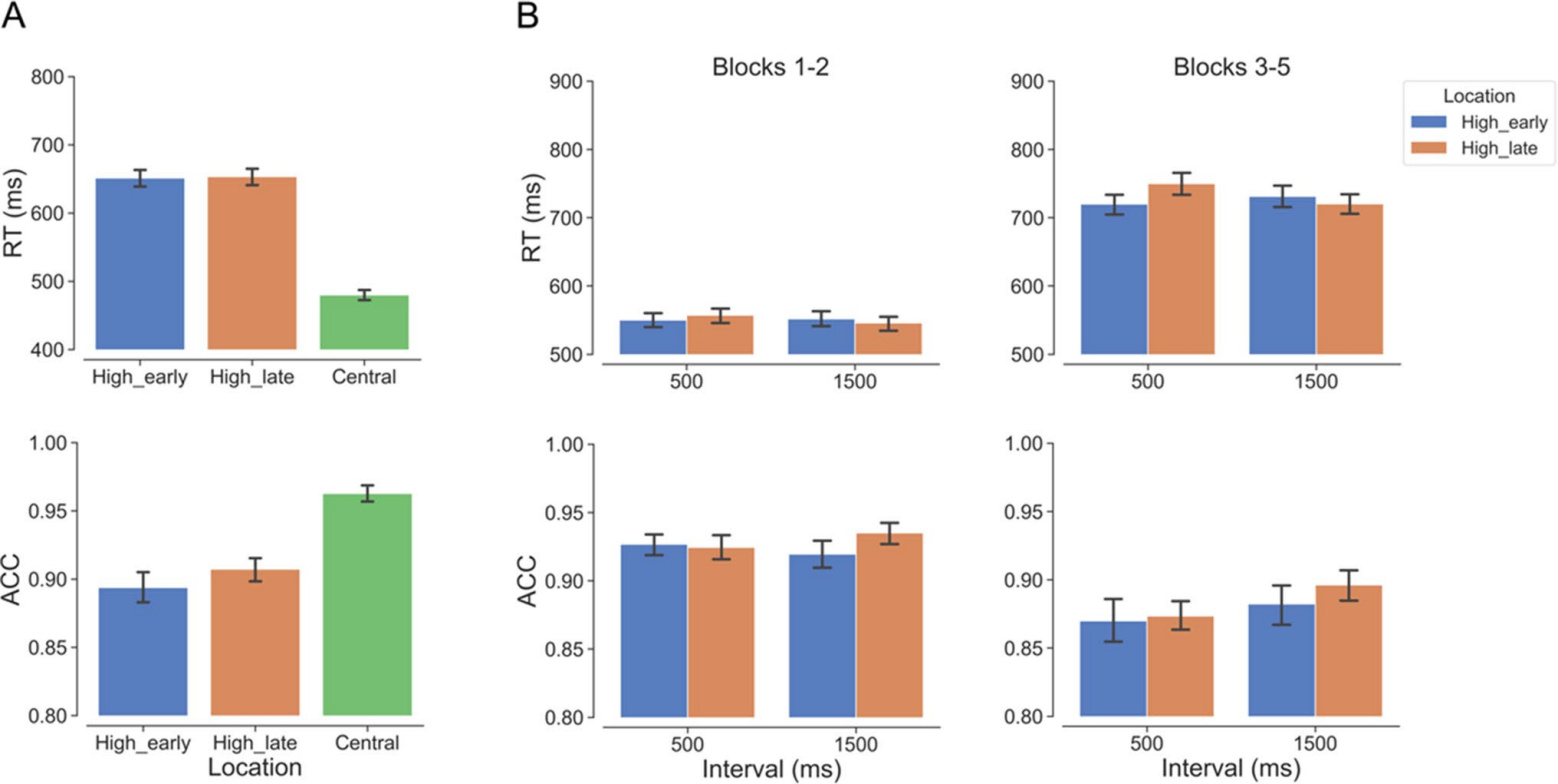
Results of Experiment 1. High_early: high probability target location at the early time point; High_late: high probability target location at the late time point; Central: target location at the center. Blocks 1–2: the two blocks with the big-gap Landolt C; Blocks 3–5: the subsequent three blocks with the small-gap Landolt C. **A** Mean RT and accuracy (ACC) as a function of target location. **B** Mean RT and ACC based on phase (Blocks 1–2, Blocks 3–5), interval (500 ms, 1,500 ms), and target location (high_early, high_late). In all graphs, error bars represent ± 1 between-subjects standard error of the condition means. (Color figure online)

As depicted in Fig. 2A, as compared with when the target appeared at the central location, participants responded slower when it appeared at either the high_early, β = 171.280, *SE* = 7.190, *t* = 23.818, *p* < 0.001, or the high_late location, β = 173.700, *SE* = 7.880, *t* = 22.036, *p* < 0.001. Also, as compared with the central location, participants showed lower accuracy for the high_early, β = − 1.281, *SE* = 0.115, *z* = − 11.091, *p* < 0.001, and the high_late location, β = − 1.195, *SE* = 0.112, *z* = − 10.662, *p* < 0.001. No significant differences were observed between the two peripheral locations in terms of RT, β = − 2.420, *SE* = 8.660, *t* = − 0.280, *p* = 0.958, or accuracy, β = − 0.086, *SE* = 0.067, *z* = − 1.289, *p* = 0.401. This central-location advantage was observed for each individual participant. These findings show that participants adhered to the instruction to initiate their search from the central location.

#### Spatiotemporal regularities in different phases

Next, we explored whether participants learned the spatiotemporal distribution of the peripheral targets in different phases of target saliency (Fig. 2B). To investigate this, we first examined the three-way interaction of interval, target location, and phase employed the (G)LMMs specified in the Data Analyses section. A significant main effect of phase was revealed. Participants responded more quicky, β = − 179.000, *SE* = 9.050, *t* = − 19.813, *p* < 0.001, and accurately, β = 0.525, *SE* = 0.084, *z* = 6.280, *p* < 0.001, in Blocks 1–2 than in Blocks 3–5. This evidence aligns with the manipulation of target saliency, as the more easily detectable stimulus (big-gap Landolt C) is the more salient one. Crucially, the results revealed a significant three-way interaction on RT, χ^2^(1) = 19.005, *p* < 0.001, but not on accuracy, χ^2^(1) = 0.654, *p* = 0.419.

Subsequently, we investigated the two-way interaction of interval and target location in both phases separately. In the analyses of accuracy, this two-way interaction was neither significant in Blocks 1–2 with salient targets, χ^2^(1) = 3.226, *p* = 0.072, nor in Blocks 3–5 with nonsalient targets, χ^2^(1) = 1.436, *p* = 0.231. In the analyses of RT, a significant interaction between interval and target location was observed in both Blocks 1–2, χ^2^(1) = 5.524, *p* = 0.019, and Blocks 3–5, χ^2^(1) = 23.806, *p* < 0.001.

The follow-up pairwise comparisons showed that, in Blocks 1–2, there was no significant difference on RT between the two peripheral locations at either the early, β = − 7.220, *SE* = 4.540, *t* = − 1.589, *p* = 0.118, or late time point, β = 5.980, *SE* = 3.930, *t* = 1.524, *p* = 0.133. In contrast, in Blocks 3–5, at the early time point, RT was significantly shorter for the temporally valid high_early location than that for the temporally invalid high_late location, β = − 30.500, *SE* = 13.700, *t* = − 2.220, *p* = 0.031. However, at the late time point, there was no significant difference between these two locations, β = − 11.600, *SE* = 13.500, *t* = − 0.858, *p* = 0.395.

In summary, the RT results revealed evidence of learning the spatiotemporal regularities during Blocks 3–5 with an early-presented nonsalient target, following two blocks of learning history with a salient target. There was also a nascent learning effect in the first two blocks, although the pairwise comparisons were not significant.

#### Awareness and intertrial priming

Based on participants’ responses to the awareness queries, 34 out of 54 participants reported noticing the variation in intervals across trials, while 20 participants did not. Regarding awareness of the spatiotemporal distribution of the target, only seven participants accurately identified the full regularities. Twenty-three participants correctly identified the high probability target location following either the short or long interval, with 15 correctly recognizing the location after the long interval and eight after the short interval. The remaining 24 participants were unable to identify any of the regularities.

The reported significant two-way interaction between interval and target location on RT, which reflects the behavioral expression of spatiotemporal regularities, was revealed after accounting for participants’ explicit awareness and intertrial priming. Derived from the LMMs testing the two-way interaction, awareness-related control factors did not significantly affect performance in either phase (see Appendix B for details). Awareness of neither the early nor late probable target location showed a significant effect, *p* values > 0.073, suggesting that top-down attention driven by awareness of regularities did not explain the observed effects.

In contrast, short-term intertrial priming significantly impacted performance apart from the significant two-way interaction (see Appendix B for details). Significant main effects were found for the repetition of target location in both phases, *p* values < 0.001. Repetition of response did not affect performance in Blocks 1–2 with salient targets, *p* = 0.731, but it significantly facilitated response speed in Blocks 3–5 with nonsalient targets, *p* = 0.040. Conversely, repetition of interval improved response speed in Blocks 1–2, *p* = 0.004, but had no effect in Blocks 3–5, *p* = 0.946. While intertrial priming influenced performance, it alone could not fully account for the observed effects.

Together, these findings suggest that participants engaged in statistical learning of the spatiotemporal regularities beyond explicit awareness or short-term priming.

#### Time course of learning spatiotemporal regularities across blocks

A three-way interaction of interval (500 ms, 1,500 ms), target location (high_early, high_late), and block number (1–5) revealed a significant effect on RT, χ^2^(4) = 24.174, *p* < 0.001, but not on accuracy, χ^2^(4) = 1.745, *p* = 0.783. Given the significant interaction on RT, we then examined the two-way interaction between interval and target location within each block (see Appendix A for details). This interaction was significant in all blocks, minimal χ^2^(1) = 3.836, *p* = 0.050 (obtained in Block 1). For the short interval, differences between high_early and high_late locations were significant in Blocks 3–5, *p* values < 0.001. In Block 1, this difference approached significance, β = − 8.400, *SE* = 4.370, *t* = − 1.924, *p* = 0.054, but was nonsignificant in Block 2, β = − 5.960, *SE* = 4.610, *t* = − 1.294, *p* = 0.196. For the long interval, location differences were only significant in Block 5, β = − 23.700, *SE* = 5.990, *t* = − 3.963, *p* < 0.001. In summary, learning effects were nascent in Blocks 1–2, but became fully evident from Block 3 onward, consistent with the phase-based analyses.

### Discussion

Experiment 1 investigated the influence of target saliency on learning interval-based spatiotemporal target regularities under two phases with salient and nonsalient target stimuli, respectively. As expected, the results confirmed that a big-gap Landolt C, with greater saliency, was detected much faster and more accurately than a small-gap one. Salient targets in peripheral locations likely drew participants’ attention, possibly evoking saccades directly toward them, resulting in faster and better detection. Nonsalient targets, on the other land, led to slower and less accurate responses, possibly due to the need for additional saccades. Despite the successful saliency manipulation, the expected facilitation of learning in Blocks 1–2 was limited, with only a nascent learning effect observed during this phase. Surprisingly, learning fully emerged in Blocks 3–5, even when targets became harder to detect. This suggests that the learning of spatiotemporal regularities continued to develop, even when attention capture by the target was diminished.

Although this finding clearly shows that spatiotemporal relations are consolidated in the second phase, even for nonsalient targets, it is not clear to what extent a prior phase with salient targets was crucial. It is possible that a first phase with salient targets is critical for learning to start off (Li et al., 2022), but it is also possible that learning would have been initiated even with nonsalient targets in the first phase. With the current findings, we could not answer the question whether the immediate capture of attention by targets was necessary to learn the interval-based spatiotemporal target regularities. Therefore, we further examined this question in another experiment in which the nonsalient target was used throughout the experiment. If the initial capture of attention indeed plays an important role in the learning that became apparent in Blocks 3–5 of Experiment 1, we should see smaller learning or no learning at all in Experiment 2, which would indicate that high target saliency is a necessary requirement for learning spatiotemporal regularities.

## Experiment 2

Experiment 2 was designed to test whether interval-based spatiotemporal target regularities could be learned even when the target was not salient. While maintaining the same spatiotemporal regularities as in Experiment 1, the small-gap Landolt C occurred in all five blocks.

### Method

Utilizing the effect size observed in the interaction of interval (500 ms, 1,500 ms) and target location (high_early, high_late) in Blocks 3–5 from Experiment 1, the 1,000 simulations indicated that a sample size of 50 participants achieves a power of 0.88 to detect the interaction effect of interest. Considering the increased difficulty of the task and potential exclusion criteria for low-performing participants, we recruited 54 students from Prolific for this experiment, ensuring a fresh pool of participants without any overlap with those of Experiment 1. After excluding one participant due to low accuracy (< 70%), the final dataset included 53 participants for subsequent analyses (*M* = 23.45 years, *SD* = 2.83, 34 women, 17 men, two other).

The apparatus, procedure and design were identical to those of Experiment 1, with the only difference being that the gap size of the Landolt C was small (2 pixels) throughout the experiment. In the data analyses, trials were excluded if the RT was below 200 ms or above 2,200 ms (0.24% of all trials). For the RT analysis, incorrect trials (7.78% of all trials) and trials with RTs outside ± 2.5 standard deviations of the condition mean of each target location for each participant (2.18% of all trials) were also excluded.

Similar to Experiment 1, we initially assessed participants’ performance at each of the three target locations. The model specifications for the (G)LMMs were consistent with those used in Experiment 1. Subsequently, we investigated whether participants learned the spatiotemporal target distribution focusing on the two peripheral locations. To compare the results to Experiment 1, we again divided the data into two phases: Blocks 1–2 and Blocks 3–5. For the (G)LMMs examining the three-way interaction of interval, target location, and phase, the model specifications remained consistent with those used in Experiment 1, with only one exception in Experiment 2. Specifically, in the (G)LMMs, the random-effect structure included a by-participant random intercept and a by-participant random slope for phase and target location. Further, we examined the two-way interaction of interval and target location for each phase with different levels of target saliency. The model specifications remained consistent with those used in Experiment 1, except that the random-effect structure of (G)LMMs featured a by-participant random intercept and a by-participant random slope for the target location.

Lastly, we investigated the time course of learning across blocks. We first examined the three-way interaction of interval (500 ms, 1,500 ms), target location (high_early, high_late), and block number (1–5) using (G)LMMs (see Appendix A for details). Following the significant three-way interaction, we investigated the two-way interaction between interval and target location within each block separately.

### Results

#### Overall performance across locations

**Fig. 3:**
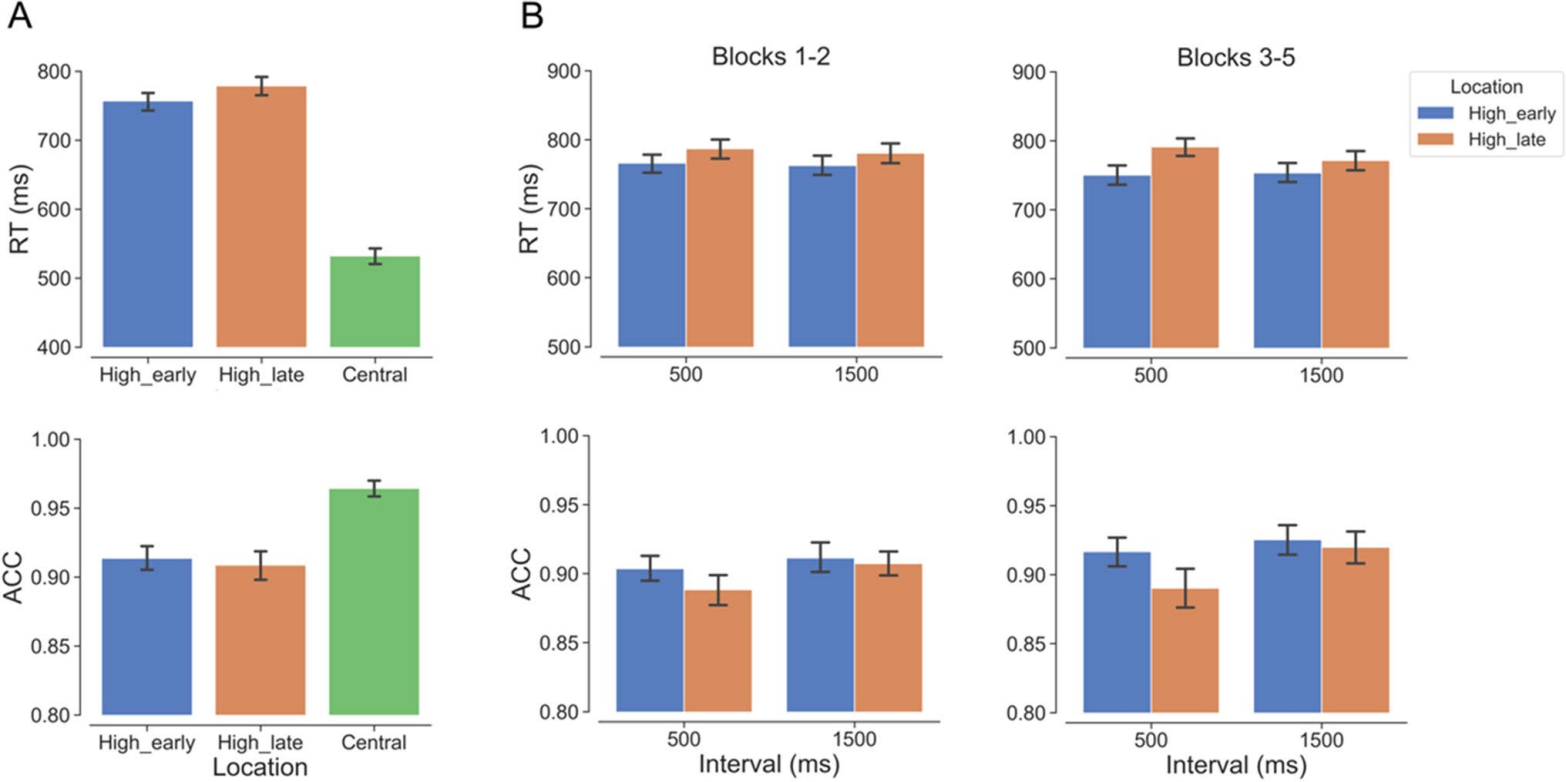
The results of Experiment 2. High_early: high probability target location at the early time point; High_late: high probability target location at the late time point; Central: target location at the center. Blocks 1–2: the first two blocks with the small-gap Landolt C; Blocks 3–5: the subsequent three blocks with the small-gap Landolt C. **A** Mean RT and ACC as a function of target location. **B** Mean RT and ACC based on phase (Blocks 1–2, Blocks 3–5), interval (500 ms, 1,500 ms), and target location (high_early, high_late). Error bars represent ± 1 between-subjects standard error of the condition means. (Color figure online)

Figure 3A shows that RT was longer when the target appeared at either the peripheral high_early, β = 224.500, *SE* = 11.090, *t* = 20.235, *p* < 0.001, or the high_late location, β = 246.400, *SE* = 9.420, *t* = 26.167, *p* < 0.001, compared with the central location. Similarly, accuracy was lower for the high_early, β = − 1.100, *SE* = 0.126, *z* = − 8.702, *p* < 0.001, and the high_late location, β = − 1.155, *SE* = 0.125, *z* = − 9.236, *p* < 0.001, compared with the central location. No notable difference was observed between the two peripheral locations in terms of either RT, β = − 21.900, *SE* = 13.750, *t* = − 1.596, *p* = 0.256, or accuracy, β = 0.055, *SE* = 0.093, *z* = 0.594, *p* = 0.823. These results indicate that participants followed the instructions of fixating and initiating the search from the central location.

#### Spatiotemporal regularities in different phases

To investigate whether participants learned the spatiotemporal target regularities across different phases, our approach mirrored that of Experiment 1 (Fig. 3B). We first examined the model that incorporated the three-way interaction of interval, target location and phase (Blocks 1–2, Blocks 3–5). Given the constant saliency throughout, there was no significant main effect of phase on RT, β = 7.360, *SE* = 5.600, *t* = 1.315, *p* = 0.194. While the accuracy in Blocks 1–2 was higher than that in Blocks 3–5, β = − 0.278, *SE* = 0.085, *z* = − 3.255, *p* < 0.01. Importantly, the analyses revealed a significant three-way interaction on RT, χ^2^(1) = 8.127, *p* = 0.004, but not on accuracy, χ^2^(1) = 0.525, *p* = 0.469.

We further investigated the two-way interaction of interval and target location in both phases separately. The analyses of RT showed that this two-way interaction was not significant in Blocks 1–2, χ^2^(1) = 0.164, *p* = 0.686, but it was significant in Blocks 3–5, χ^2^(1) = 11.686, *p* < 0.001. In Blocks 3–5, the follow-up pairwise comparisons showed that, at the early time point, RT was significantly shorter for the temporally valid high_early location than that for the temporally invalid high_late location, β = − 41.400, *SE* = 14.700, *t* = − 2.824, *p* = 0.007. However, at the late time point, there was no significant difference between the two locations, β = − 17.200, *SE* = 14.700, *t* = − 1.175, *p* = 0.245.

In the analyses of accuracy, the two-way interaction between interval and target location was not significant in Blocks 1–2, χ^2^(1) = 0.838, *p* = 0.360, but it was significant in Blocks 3–5, χ^2^(1) = 4.925, *p* = 0.026. In Blocks 3–5, the follow-up pairwise comparisons showed that, at the early time point, accuracy was significantly higher for the temporally valid high_early location than that for the temporally invalid high_late location, β = 0.293, *SE* = 0.133, *z* = 2.204, *p* = 0.028. However, at the late time point, there was no significant difference between the two locations, β = 0.024, *SE* = 0.140, *z* = 0.169, *p* = 0.866.

In summary, the results revealed evidence of learning the spatiotemporal regularities during Blocks 3–5 after the short interval, following two blocks with a nonsalient target. Although there was no evidence for learning the spatiotemporal relationship during the first two blocks, learning might have started off there in view of the learning effects observed in Blocks 3–5.

#### Awareness and intertrial priming

Out of 53 participants, 36 reported noticing the interval variations across trials, while 17 did not. When asked to identify the most probable target location following both the short and long intervals, only seven participants successfully reported the complete regularities. Nineteen participants identified the regularities for either the short or long interval, with eight correctly reporting the probable target location after the long interval and 11 after the short interval. The remaining 27 participants were unable to report any of the regularities.

In the analyses of the two-way interaction between interval and target location in Blocks 3–5, where the behavioral expression of spatiotemporal regularities was observed, participants’ awareness had minimal influence on performance. There were no significant effects of awareness-related factors on either RT or accuracy (*p* values ≥ 0.108), except for a notable increase in accuracy due to awareness of the late probable location, *p* = 0.034 (see Appendix A for details).

In contrast, short-term intertrial priming substantially impacted participants’ performance apart from the significant two-way interaction (see Appendix A for details). In Blocks 3–5, where learning effects emerged, repetition of target location significantly enhanced performance on both RT and accuracy, *p* values < 0.001. Intertrial repetition of the interval facilitated RT, *p* < 0.001, but not accuracy, *p* = 0.236. Meanwhile, repetition of the response had no significant effect on RT, *p* = 0.182, but lead to a reduction in accuracy, *p* = 0.002.

In summary, the behavioral expression of spatiotemporal regularities persisted even after accounting for awareness and intertrial priming, suggesting that participants’ performance improvements were not solely due to their explicit awareness of the regularities or to short-term priming, but rather to statistical learning of the spatiotemporal regularities.

#### Time course of learning spatiotemporal regularities across blocks

To explore the development of learning over time, we examined the three-way interaction of interval (500 ms, 1,500 ms), target location (high_early, high_late), and block number (1–5). A significant interaction was found for RT, χ^2^(4) = 13.741, *p* = 0.008, but not for accuracy, χ^2^(4) = 1.769, *p* = 0.778. Given this significant interaction on RT, we analyzed the two-way interaction between interval and target location within each block (details in Appendix A). This interaction was not significant in Block 1 and 2, *p* values ≥ 0.543, became highly significant in Block 3 and 4, *p* values < 0.001, but was no longer significant in Block 5, χ^2^(1) = 1.878, *p* = 0.171. Pairwise comparisons for Blocks 3 and 4 showed that after the short interval, RTs were shorter for the high_early location compared with high_late location, *p* values ≤ 0.018, while no significant difference was found after the long interval, *p* values ≥ 0.458. In summary, learning effects were minimal in the early Blocks 1–2, reached a peak in Blocks 3 and 4, and diminished again in Block 5.

#### The comparison of learning effects between experiments

To compare the observed learning effects on RT between experiments, we focused on Blocks 3–5, which showed the strongest expressions of learning. For each interval, we calculated the RT difference between temporally invalid and temporally valid target locations, using these differences as indices of learning magnitude. Independent *t* tests were then conducted to examine the effect of Experiment (EXP1, EXP2) on these learning magnitudes. Bayes factors were computed using JASP (Version 0.18.3) with a default prior distribution (JASP Team, 2024) to quantify evidence in favor of the null hypothesis over the alternative hypothesis (BF01).

The results indicated that at the early time point, where the most pronounced learning effect was observed, the learning effect was comparable between Experiment 1 (*M* = 30.024, *SE* = 101.042) and Experiment 2 (*M* = 40.661, *SD* = 106.591), *t*(105) = − 0.530, *p* = 0.597, *d* = − 0.102, BF01 = 4.310. Similarly, at the late time point, the learning effect was also comparable between Experiment 1 (*M* = 10.971, *SD* = 99.999) and Experiment 2 (*M* = − 17.866, *SD* = 108.325), *t*(105) = 1.431, *p* = 0.155, *d* = 0.277, BF01 = 1.962. Overall, the evidence suggest that learning was comparable between Experiment 1 and Experiment 2 in Blocks 3–5, indicating that the initial exposure to target saliency did not significantly influence the magnitude of learning observed during subsequent nonsalient target conditions.

### Discussion

Experiment 2 examined the learning of interval-based spatiotemporal target regularities with nonsalient targets throughout the experiment. With the same target saliency in the two phases, there was no apparent difference in response speed between Blocks 1–2 and Blocks 3–5. Still, we obtained evidence that spatiotemporal regularities were learned. When comparing performance between the two phases, Experiment 2 replicated the general pattern observed in Experiment 1, although no evidence was obtained for learning in the first two blocks. However, some learning might have taken place even in this phase since a clear learning effect was apparent in Blocks 3–5. That is, despite the absence of initial attentional capture by the targets, participants adapted to the spatiotemporal target regularities during visual search without explicit awareness. Also, similar to Experiment 1, the typical finding of learning spatiotemporal regularities came to expression at the early interval but not at the late one, which is a typical observation in similar designs (Xu et al., 2021, 2023). Furthermore, direct comparison between experiments revealed equivalent learning in Blocks 3–5, regardless of whether participants had initially been exposed to salient targets (Experiment 1) or to nonsalient targets (Experiment 2). Taken together with the findings of Experiment 1, these findings indicate that target saliency is not only unnecessary for maintaining learned spatiotemporal regularities but also not for learning them in the first place.

## General discussion

The current study investigated whether target saliency is a prerequisite for learning interval-based spatiotemporal target regularities. The findings provide clear evidence that the learning of distributional spatiotemporal target regularities can occur in the absence of target saliency. In Experiment 2, where targets remained nonsalient throughout, participants showed the capacity to learn the spatial distribution of targets unfolding in time, particularly at the early time point. When comparing the learning effects between two experiments, the results revealed that the initial exposure to salient targets did not lead to a subsequent stronger expression of the spatiotemporal regularities. This evidence suggests that the learning of spatiotemporal target regularities operates independently of target saliency. Together, these findings uncovered a resilient learning mechanism in adapting to spatiotemporal target regularities, irrespective of saliency conditions.

Meanwhile, the results are highly consistent with previous studies with salient targets in more complex displays (Xu et al., 2023). Note that in all these studies, the learning effect is quite small and only evident in the early time point but much less so in the late time point. Despite design measures aimed at enhancing effect size, such as simplifying the display, requiring eye movements toward the target, and reducing exposure duration, the small asymmetric benefit pattern persisted in the current study. This raises intriguing question about its underlying mechanisms. In the additional singleton task, where all the items were visible while fixating, it was previously assumed that activation or suppression of the high probability location in the early time point would persist until the late time point (Xu et al., 2021, 2023). However, in the current task, with distant peripheral locations, nonsalient targets, and brief display presentation, detecting the peripheral target would be challenging if attention was initially misdirected to the other peripheral location. In such cases, optimizing search efficiency should have involved direct focusing on the temporally valid high_late location. The persistence of the asymmetric benefit pattern in our Landolt C search task, particularly at two remote locations, suggests that factors beyond simple time–location contingency learning may be at play. Furthermore, given the observed asymmetric learning effect, future studies should include a temporally neutral condition like in Xu et al., (2021, 2023) to establish whether learning is genuinely tied to time–location contingencies or driven by a general bias for the early time point. This would enable direct comparisons between performance at late high probability and neutral locations, allowing the examination of possible learning effects at late time point that we may have missed in the current design.

It is noteworthy that the current demonstration of spatiotemporal target regularities learning excluded a mediating role of the manual motor response. In the paradigm used, the feature relevant to the spatiotemporal regularity (left–right) was separated from the response defining feature (up-down). Therefore, going beyond Boettcher et al. (2022), the current findings demonstrated VSL of interval-based spatiotemporal target regularities in the absence of both target saliency and manual motor response learning. However, it must be acknowledged that the current design does not exclude the potential involvement of saccade movement learning. In the previous study (Xu et al., 2023), participants could covertly search items while maintaining central fixation. However, in the current study with peripheral locations, participants were likely to identify the peripheral target by moving their eyes towards it. In essence, participants also needed to learn to direct their gaze for effective searching. Therefore, we cannot rule out the possibility that saccade movements constitute an important component in the learning process.

Given the evidence for learning in the absence of target saliency, the current findings stand in stark contrast to learning the spatiotemporal target regularities concerning cross-trial sequential associations of target locations. In that field, research has consistently shown that intertrial transitional regularities of target locations are predominantly learned when the target conspicuously pops out from the surroundings either in both the predicting trial and the predicted trial (Li & Theeuwes, 2020; Li et al., 2022; Ono et al., 2005; Yu et al., 2023), or at least in the predicting trial (Ono et al., 2005). These sequence-based transitional spatiotemporal target regularities focus on sequential dependencies of target locations between adjacent trials, making them more susceptible to noise or disruptions in the sequence (Ono et al., 2005). The absence of a pop-out target may enforce a slow serial deployment of attention to each item in the display until the target is found, thereby obscuring intertrial regularities. In comparison, the presence of a pop-out target may have helped participants to overcome the interference generated by attending nontargets, thus promoting the learning of cross-trial regularities, resulting in better performance (Li & Theeuwes, 2020).

One can think of several reasons why noise would have a lesser impact on the detection of within-trial spatiotemporal target regularities, such as those of the present study. One reason could be that a trial constitutes a natural demarcation of an experienced episode (Logan, 1988, 1990; Los et al., 2014, 2021). As a result, within-trial dependencies are stored more robustly in memory than between-trial dependencies, so they come more readily to expression in behavior. Another reason could be that within-trial regularities extend over a shorter time frame than between-trial regularities, such that the representations involved in the regularities can be mutually related more easily. In either case, learning would be less susceptible to noise, thereby reducing the relevance of target saliency.

In conclusion, the current study indicates that target saliency is not a necessary condition for learning the dynamic spatial distribution of targets unfolding in time. The learning effects were equally strong whether the initial phase involved a highly salient target or a nonsalient one that did not capture attention. This suggests that attentional guidance of target search is a critical factor for the creation of memory representations that underlie the learning process.

## Supplementary Information

Below is the link to the electronic supplementary material.

Supplementary file1 (DOCX 5.60 KB)

## Data Availability

The raw data, processed data, and data for power analysis are available on the Open Science Framework (https://osf.io/vkn6a/).

## Code availability

The experimental programs, analysis scripts, and power analysis code are available on the Open Science Framework (https://osf.io/vkn6a/).

## Declarations

### Open practices statement

The experimental programs, raw data, processed data, analysis scripts, and power analysis code are available on the Open Science Framework (https://osf.io/vkn6a/). None of the experiments was preregistered.
