# Supplementary material for: Statistical learning of spatiotemporal target regularities in the absence of saliency
Source: Atten Percept Psychophys. 2025 Jan 9;87(2):431–44. doi: 10.3758/s13414-024-02992-6 (PMC11865177; doi:10.3758/s13414-024-02992-6)
Supplement: Supplementary file 1 — Supplementary file1 (DOCX 5.60 KB) [file 13414_2024_2992_MOESM1_ESM.docx]

**Appendix:**

Statistical learning of spatiotemporal target regularities in the absence of saliency

Zhenzhen Xu, Jan Theeuwes & Sander A. Los

Department of Experimental and Applied Psychology,

Vrije Universiteit Amsterdam, Amsterdam, The Netherlands

Institute Brain and Behavior Amsterdam (iBBA), Amsterdam, The Netherlands

z.z.xu@vu.nl

**Appendix A. Time course of learning spatiotemporal regularities across blocks**

**Experiment 1**

We conducted a block-by-block analysis to investigate the time course of learning across blocks, acknowledging the potential limitations in statistical power. First, we examined the three-way interaction of interval (500 ms, 1500 ms), target location (high_early, high_late), and block number (1-5) using (G)LMMs. The models included fixed effects of interval, target location, block number, their three-way interaction, and all the control fixed-effects factors. In the LMMs, the random-effect structure comprised a by-participant random intercept and a by-participant random slope for the interval, target location, and block number. In the GLMMs, the random-effect structure comprised a by-participant random intercept. The results revealed a significant three-way interaction on RT, χ^2^ (4) = 24.174, *p* < .001, but not on accuracy, χ^2^ (4) = 1.745, *p* = .783.

Following the significant three-way interaction on RT, we then investigated the two-way interaction between interval and target location within each block separately. The LMMs incorporated fixed effects of interval, target location, their interaction, and control factors. The random-effect structure of the LMMs comprised a by-participant random intercept and a by-participant random slope for the interval.

As shown in Figure 4, the analyses of RT showed that the interaction between interval and target location became significant in block 1, χ^2^ (1) = 3.836, *p* = .050. Pairwise comparisons indicated that at the early time point, the difference between high_early and high_late locations approached significance, β = -8.400, *SE* = 4.370, *t* = -1.924, *p* = .054, but not at the late time point, β = 3.690, *SE* = 4.360, *t* = 0.846, *p* = .398. In block 2, this two-way interaction was significant, χ^2^ (1) = 4.679, *p* = .031. However, pairwise comparisons revealed no significant differences between high_early and high_late locations at either the early time point, β = -5.960, *SE* = 4.610, *t* = -1.294, *p* = .196, or the late time point, β = 8.140, *SE* = 4.610, *t* = 1.765, *p* = .078. In block 3, the interaction between interval and target location became highly significant, χ^2^ (1) = 12.630, *p* < .001. Pairwise comparisons showed a significant benefit for the temporally valid high_early location over the temporally invalid high_late location at the early time point, β = -29.665, *SE* = 6.070, *t* = -4.883, *p* < .001. In contrast, there was no significant difference between two locations at the late time point, β = 0.767, *SE* = 6.030, *t* = 0.127, *p* = .899. In block 4, the significant two-way interaction persisted, χ^2^ (1) = 27.020, *p* < .001. Pairwise comparisons again revealed a significant difference at the early time point, β = -34.900, *SE* = 6.090, *t* = -5.727, *p* < .001, but not at the late time point, β = 9.810, *SE* = 6.050, *t* = 1.620, *p* = .105. In the last block 5, the interaction remained highly significant, χ^2^ (1) = 40.407, *p* < .001. Pairwise comparisons reveled that, at the early time point, RT was significantly shorter for the temporally valid high_early location compared to the temporally invalid high_late location, β = -30.700, *SE* = 6.100, *t* = -5.034, *p* < .001. At the late time point, RT was also significantly shorter for the temporally valid high_late location compared to the temporally invalid high_early location, β = -23.700, *SE* = 5.990, *t* = -3.963, *p* < .001.

**Experiment 2**

We first examined the three-way interaction of interval (500 ms, 1500 ms), target location (high_early, high_late), and block number (1-5) using (G)LMMs, with model specifications consistent with those used in Experiment 1. The results revealed a significant three-way interaction on RT, χ^2^ (4) = 13.741, *p* = .008, but not on accuracy, χ^2^ (4) = 1.769, *p* = .778. Following the significant three-way interaction on RT, we investigated the two-way interaction between interval and target location within each block separately using LMMs. The random-effect structure including a by-participant random intercept and a by-participant random slope for the interval and target location.

The RT analyses (Figure 4) showed that the interaction between interval and target location was neither significant in block 1, χ^2^ (1) = 0.370, *p* = .543, nor in block 2, χ^2^ (1) = 0.004, *p* = .949. However, in block 3, this interaction became highly significant, χ^2^ (1) = 19.958, *p* < .001. Pairwise comparisons indicated a significant benefit for the temporally valid high_early location over the temporally invalid high_late location at the early time point, β = -48.200, *SE* = 15.800, *t* = -3.055, *p* = .003, with no significant difference between locations at the late time point, β = -11.800, *SE* = 15.700, *t* = -0.747, *p* = .458. The significant two-way interaction persisted in block 4, χ^2^ (1) = 11.014, *p* < .001, with a significant difference at the early time point, β = -36.920, *SE* = 15.100, *t* = -2.440, *p* = .018, but not at the late time point, β = -9.240, *SE* = 15.100, *t* = -0.612, *p* = .543. In block 5, the interaction was not significant, χ^2^ (1) = 1.878, *p* = .171.

**Figure 4**

*Time course of learning spatiotemporal regularities across blocks in RT*

*
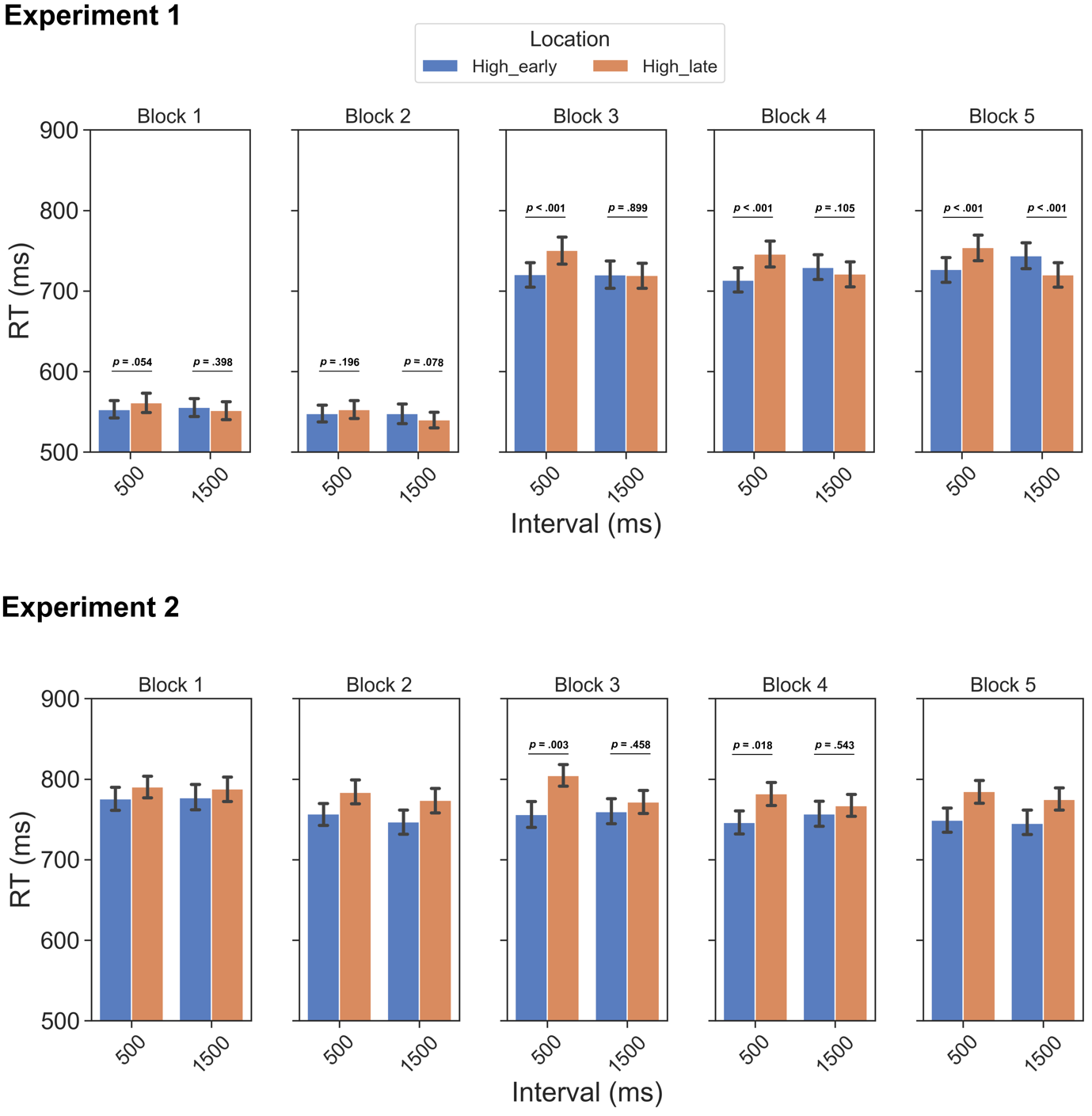
*

*Note.* Mean RT as a function of Block (Block 1-5), interval (500 ms, 1500 ms), and target location (high_early, high_late) in Experiments 1 and 2. High_early: high probability target location at the early time point; High_late: high probability target location at the late time point (high_late). Blocks 1 and 2: the blocks with the big-gap Landolt C in Experiment 1 and the blocks with the small-gap Landolt C in Experiment 2; Blocks 3, 4, and 5: the blocks with the small-gap Landolt C in both Experiments 1 and 2. The *p* values for pairwise comparisons were only noted in blocks showing significant two-way interaction between interval and target location. Error bars represent ± 1 between-subjects standard error of the condition means.

**Appendix B. The effect of awareness and intertrial priming**

**Experiment 1**

In the RT analyses of the two-way interaction between interval and target location, where the behavioral expressions of spatiotemporal regularities were observed, participants’ awareness did not significantly influence performance. Specifically, in blocks 1-2, neither awareness of the early probable location, β = -39.312, *SE* = 21.486, *t* = -1.830, *p* = .073, nor awareness of the late probable location, β = 12.268, *SE* = 19.589, *t* = 0.626, *p* = .534, significantly affected performance. Similar findings were found in blocks 3-5, where the learning effect was most pronounced: awareness of neither the early probable location, β = 16.380, *SE* = 28.266, *t* = 0.580, *p* = .565, nor the late probable location, β = 10.345, *SE* = 25.772, *t* = 0.401, *p* = .690, had a significant impact on performance. These results suggest that the observed effect was not due to top-down modulation of attention driven by participants’ awareness of the regularities.

In contrast, factors related to short-term intertrial priming significantly influenced participants’ performance. In blocks 1-2, the repetition of target location, β = -23.292, *SE* = 2.048, *t* = -11.374, *p* < .001, and interval, β = -5.762, *SE* = 2.005, *t* = -2.873, *p* = .004, significantly facilitated response speed, whereas the repetition of response did not, β = 0.675, *SE* = 1.962, *t* = 0.344, *p* = .731. In blocks 3-5, the repetition of target location, β = -36.634, *SE* = 2.157, *t* = -16.981, *p* < .001, and response, β = -4.250, *SE* = 2.072, *t* = -2.051, *p* = .040, significantly facilitated response speed, but the repetition of interval did not, β = 0.143, *SE* = 2.120, *t* = 0.068, *p* = .946.

**Experiment 2**

In the analyses of the two-way interaction between interval and target location in blocks 3-5, where the behavioral expressions of spatiotemporal regularities were observed, participants’ awareness did not significantly influence performance. On RT, neither awareness of the early probable location, β = 13.035, *SE* = 23.990, *t* = 0.543, *p* = .589, nor awareness of the late probable location, β = -41.188, *SE* = 25.219, *t* = -1.633, *p* = .108, significantly affected performance. On accuracy, where the learning effect was most pronounced for early presented targets, awareness of the early probable location did not significantly impact participants’ performance, β = 0.400, *SE* = 0.277, *z* = 1.442, *p* = .149. Only the awareness of the late probable location increased participants’ performance, β = 0.593, *SE* = 0.279, *z* = 2.123, *p* = .034.

In contrast, factors related to intertrial priming significantly influenced participants’ performance. In blocks 3-5, where the learning effect was most pronounced, the repetition of target location, β = -45.548, *SE* = 2.176, *t* = -20.933, *p* < .001, and interval, β = -7.228, *SE* = 2.134, *t* = -3.388, *p* < .001, significantly facilitated response speed, but the repetition of response did not, β = -2.792, *SE* = 2.090, *t* = -1.336, *p* = .182. On accuracy, the repetition of target location significantly improved performance, β = 0.338, *SE* = 0.056, *z* = 5.988, *p* < .001, but not interval repetition, β = 0.063, *SE* = 0.053, *z* = 1.184, *p* = .236. Surprisingly, the repetition of response reduced the accuracy, β = -0.163, *SE* = 0.053, *z* = -3.100, *p* = .002.
